# Evaluation of the Tasso+ blood self-collection device for quantitation of plasma cytomegalovirus (CMV) DNAemia in adult solid organ transplant recipients (SOTr)

**DOI:** 10.1128/spectrum.00030-24

**Published:** 2024-05-21

**Authors:** Tran Phan, Lakshin Kumar, Marissa Woo, Alicja Sadowska-Klasa, Jared Castor, Greg Pepper, Cynthia E. Fisher, Ajit P. Limaye

**Affiliations:** 1 Division of Allergy and Infectious Diseases, Department of Medicine, University of Washington, Seattle, Washington, USA; 2 Vaccine and Infectious Disease Division, Fred Hutchinson Cancer Center, Seattle, Washington, USA; 3 Department of Hematology and Transplantology, Medical University of Gdansk, Gdansk, Poland; 4 Department of Lab Medicine and Pathology, University of Washington, Seattle, Washington, USA; University of California, San Diego, La Jolla, California, USA

**Keywords:** cytomegalovirus, virology, transplantation, clinical methods

## Abstract

Quantitative monitoring of cytomegalovirus (CMV) DNAemia in venous blood is standard in solid organ transplant recipients (SOTr) but is limited by the need for phlebotomy facilities and personnel. The aim of the study was to evaluate the Tasso+ capillary blood (CB) self-collection device for quantitation of plasma CMV DNAemia. Thirty adult SOTr with suspected CMV DNAemia were enrolled to have a supervised Tasso+ CB sample collection within 24 h of a venous sample. CMV DNA was quantitated in paired samples by using the Abbott M2000 Real-Time qPCR instrument. The participants were provided with a study-specific survey that measured patient acceptability of the Tasso+ device compared with venipuncture. A Tasso + CB sample was successfully collected in 28/30 (93%) patients, and 44 paired samples were analyzed. Concordance for detection of CMV DNAemia above the limit of detection (LOD) was 91% (42/44), and the Tasso + CB sample was estimated to be 95% sensitive at a viral load (VL) of 308 IU/mL. Among samples with a quantifiable DNAemia result with both methods (*N* = 31), there was excellent correlation between methods (Spearman R^2^ = 0.99). The difference in CMV VL between venous and Tasso+ CB samples was not dependent on time (*P* > 0.1). Of 12 who completed the survey, 11 (92%) expressed a preference for Tasso+ CB collection over venipuncture. Collection of CB with the Tasso+ device is feasible, patient-acceptable, and yields generally comparable CMV DNAemia load to standard venous samples, but with lower sensitivity. Future studies to optimize and evaluate this methodology for patient self-collected samples are warranted.

**IMPORTANCE:**

We evaluate an FDA-cleared blood self-collection device (Tasso+) and demonstrate that it is patient-acceptable and yields a liquid blood sample with quantitative CMV DNAemia results comparable to those of standard venipuncture samples. This opens up possibilities for self-blood collection to monitor for CMV and potentially other viruses in transplant and other at-risk populations.

## INTRODUCTION

Cytomegalovirus (CMV) is one of the most common viral infections in transplant recipients, and quantitative assessment of CMV DNAemia has a well-established clinical utility to guide initiation of an antiviral drug in preemptive therapy protocols, diagnose CMV disease, and assess response to treatment (1). Measurement of CMV DNAemia is typically performed using venous blood samples. However, venipuncture is limited by the need for phlebotomy-trained personnel and facilities, patient acceptability, logistical issues (need for transportation to facility, wait times), and concerns regarding access for those with transmissible infections (as learned during the COVID-19 pandemic). Blood self-collection approaches could address some of these concerns in stustandard phlebotomy.

Capillary blood (CB) collection approaches, such as dried blood spots, have been previously studied for CMV DNAemia quantitation but are limited by patient acceptability and the lack of a liquid sample for optimal quantitative analyses. The Tasso+ device, through the use of microneedle technology, allows for self-collection of a liquid CB sample with a minimally invasive and relatively simple process (2, 3). Recent studies have shown good concordance between samples of Tasso+ device-collected CB and phlebotomy venous samples for the measurement of several infectious disease-related analytes, including SARS-CoV-2 antibodies (4, 5), HIV copy number, syphilis antibodies, and creatinine levels (6). However, there are no prior published studies that systematically compare Tasso+ CB to standard venous samples for quantitative measurement of plasma CMV DNAemia.

The aim of this study was to assess the feasibility, patient acceptability, and concordance of plasma CMV DNAemia quantitation between Tasso+ CB and standard venous blood samples.

## MATERIALS AND METHODS

### Patients and samples

This was a prospective, institutional review board-approved study (UW IRB#00011835) conducted at a single academic U.S. transplant center with a large multi-organ transplant program. All participants signed informed consent before study procedures. Potential study participants were identified through the electronic health record by screening for SOTr who had CMV DNAemia assessed as part of routine clinical care (via standard phlebotomy venipuncture). Those with CMV DNAemia and who were scheduled for future CMV DNAemia testing were approached for study participation at their next clinically scheduled blood draw (typically within 1 week of their prior blood draw). Inclusion criteria were age ≥18 years, able to provide informed consent, receipt of a SOT and receiving immunosuppression, plasma CMV DNAemia detected during clinical testing, planned follow-up clinical CMV DNAemia testing at the medical center, and anticipated to have detectable plasma CMV DNAemia during the next scheduled clinical blood draw. Exclusion criteria were previous participation in a study of the Tasso+ device or inability to have a Tasso+ CB sample collected within 24 h of the venous sample collection. The patients were enrolled during their outpatient phlebotomy visit or in their hospital room if they were hospitalized.

Commercially available Tasso+ devices were purchased from the manufacturer. CB samples were collected using the Tasso+ device according to the manufacturer’s instructions (7). The participants were offered the option to either perform the blood collection by themselves (“self-collected”) in the presence of study personnel or for the research team member to collect the sample (“research coordinator [RC]-collected”). Thermal pads (Medline MDS139009) were placed over the upper arm for 5 min before device placement to optimize regional blood flow. The Tasso+ device was then placed on the upper arm. The device was activated by pressing the button to activate the microneedle system and kept on the arm until either the collected blood volume was approximately 500 μL or for 10 min, whichever occurred first. The tubes were centrifuged immediately after collection, and plasma was transferred to cryovials and stored at −80°C until analysis. At the time of batch testing, the samples were thawed and diluted with phosphate buffered saline to a final volume of 700 μL. Venipuncture was performed according to standard procedures using 5 mL EDTA-containing blood tubes. For both sample types, the commercially available Abbot M2000sp was used for sample nucleic acid extraction, and the Abbot M2000rt qPCR assay was used for CMV DNAemia quantitation in plasma. The limit of detection (LoD) for this assay was 30 IU/mL (1.47 $\log_{10}\frac{IU}{mL}$), and the lower limit of quantitation (LLoQ) was 50 IU/mL (1.69 $\log_{10}\frac{IU}{mL}$).

The participants were sent a five-question study-specific survey assessing patient acceptability after completion of the study visit (see the supplemental material). The first three questions assessed the discomfort of venous sample and Tasso+ methods and the ease of use of the Tasso+ device on a numeric scale of 1–5. The last two questions assessed confidence in using the Tasso+ device and preference of method for future blood draws using the Likert scale.

### Statistical analyses

Concordance in CMV DNAemia measurements between methods was assessed both categorically and quantitatively. Agreement was calculated for classification into the categories of not detected, detected but <LLoQ, and detected >LLoQ. To correct for the probability of chance agreement, categorical concordance was also measured using Gwet’s AC1 for detection and quantitation; this method is less affected by bias compared with Cohen’s kappa. Sensitivity of the Tasso+ device method was assessed for detection >LoD (vs. <LoD) and for detection >LLoQ. Formally, sensitivity is typically expressed as the lowest viral load expected to test positive in 95% of replicates. However, since this was not feasible for the current study, we estimated the sensitivity of the Tasso+ CB blood samples using probit regression as previously reported in a study with a similar study design (8). Concordance in quantitative CMV DNAemia (viral load [VL]) between methods was assessed among the subset of samples that yielded a quantifiable result by both methods. Scatter plots and linear regression were used to identify whether a linear correlation existed between the two methods. Agreement was then assessed using Bland–Altman plots (9, 10). The limits of agreement were compared to an *a priori* clinically acceptable difference (CAD) of $\pm 0.5\;log_{10}\frac{IU}{mL}.$ The limits of agreement were calculated using a nested method that accounts for within- and between-subject variations (11). The concordance correlation coefficient (CCC) was also calculated for quantitated values (12). Concordance between sequential samples was assessed visually by comparing the mean difference in CMV VL at the first and last samples and creating a linear model regressing the difference in CMV VL on time after study enrollment. Patient acceptability was assessed from survey responses using descriptive analyses.

## RESULTS

### Patients and samples

**Fig 1:**
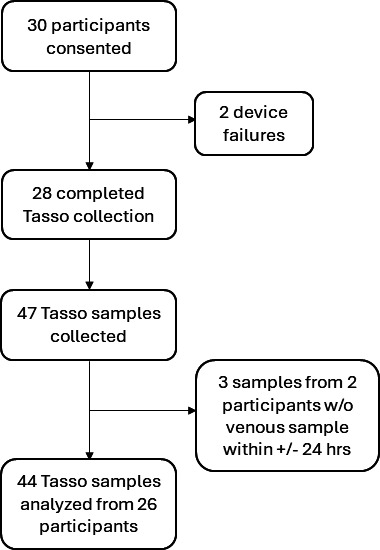
Flow of participants and samples.

Initially, 30 patients met the eligibility criteria and provided informed consent. A Tasso+ CB sample was successfully collected in 28/30 (93%) participants and 47/49 (96%) individual sample collections. Of these 47 samples, 3 (6%) were excluded because the time difference between the Tasso+ CB and venous sample collections was >24 h, leaving 44 paired samples from 26 patients available for analysis (Fig. 1). Table 1 shows the characteristics of the study population. Of those who had at least one analyzable Tasso+ CB sample collected, seven participants (16 samples) self-collected the sample under RC supervision, and 19 participants (28 samples) had the sample collected by the RC. The mean, median, and range of CB volume were 0.59, 0.55, and 0.15–1.0 mL, respectively, and did not differ significantly between self-collected vs. RC-collected specimens. Of the 26 patients analyzed in the study, 11 (39%) patients were diagnosed with CMV disease during at least one of their sample collections, with 16/44 (34%) samples being collected during an episode of CMV disease. One patient further developed CMV disease after study completion. Moreover, 24/26 (92%) participants were receiving CMV therapy at the time of enrollment/sample collection. Among the 44 sample pairs collected, 42 (95%) were collected during anti-CMV therapy that included valganciclovir (*N* = 19), intravenous ganciclovir (*N* = 18), maribavir (*N* = 4), or intravenous foscarnet (*N* = 1).

**TABLE 1:** Patient demographics (*n* = 30)

Parameter	Result
Age in years at enrollment (median [IQR])	61.2 [16.8]
Gender	
Male	21
Female	9
CMV serostatus	
D−R−	0
D+R−	25
D+R+	4
D−R+	1
Time post-transplant in months (median [IQR])	5.1 [2.1–7.8]
Weight in kg (mean ± SD)	83.3 ± 21.3
Transplant type	
Liver	12
Heart	8
Lung	5
Kidney	2
Kidney/heart	1
Kidney/lung	1
Heart/lung	1

### Categorical agreement for detection and quantitation of plasma CMV DNAemia between venous and Tasso+ methods

Categorical concordance between methods for the dichotomous categories of CMV DNA detected (>LoD) vs no CMV DNA detected (<LoD) was high with a percent agreement of 91% (40/44) and Gwet’s AC1 of 0.89. Agreement between methods using a three-category analysis (<LoD, >LoD but <LLoQ, or >LLoQ) was lower than for a two-category dichotomous analysis, with a percent agreement of 77% and Gwet’s AC1 of 0.70. Among the 11 samples for which there was category discordance (Table 2), the dilution factor, calculated as the final volume divided by the volume of the initial sample, was numerically higher compared with the rest of the concordant result samples (7.73 vs 5.50) but not significantly different (*P* > 0.1). The median (IQR) viral load in sample pairs for which the venous sample was >LLoQ, but the Tasso+ CB result not detected (<LoD) was 116 (98–151) IU/mL compared with a median (IQR) CMV VL of 1917 (436–11,093) IU/mL in samples in which CMV DNAemia was detected in Tasso+ CB. The proportion of discordant samples was not different between self-collected vs RC-collected samples (0.19 vs 0.29, Fisher’s exact test, *P =* 0.72). CMV DNAemia ≥1000 IU/mL was detected in 23 venous samples, with a median (IQR) CMV VL of 7270 (3680–18,737) IU/mL. CMV DNAemia ≥1000 IU/mL was identified in 22/23 (96%) of the corresponding Tasso+ CB samples with a median (IQR) CMV VL of 7400 (2652–76,812). In the one discordant sample pair, the CMV VL values were 1000 and 650 IU/mL in the venous and Tasso+ CB samples, respectively.

**TABLE 2:** Summary of samples

Tasso+ CB	Venous sample		
	Not detected	Detected below LLoQ (≥ LOD)	Detected and quantitated (≥ LLoQ)
Not detected	2	1	3
Detected below LLoQ (≥ LOD)	0	0	7
Detected and quantitated (≥ LLoQ)	0	0	31

### Sensitivity and specificity

To assess sensitivity, we regressed the probability of detection >LLoQ in the Tasso+ CB sample as a function of the CMV DNAemia level in the venous sample. We identified a 95% probability of detection of CMV DNAemia >LoD in the Tasso+ CB sample at a threshold of 308 IU/mL in the plasma from the venous sample. For a 95% probability of detection >LLoQ in the Tasso+ CB sample, the corresponding threshold in the plasma from the venous sample was approximately 588 IU/mL. There were no false positives in the Tasso+ CB samples, so the specificity was 1.

### Agreement between CMV viral loads in Tasso+ CB and venous samples

**Fig 2:**
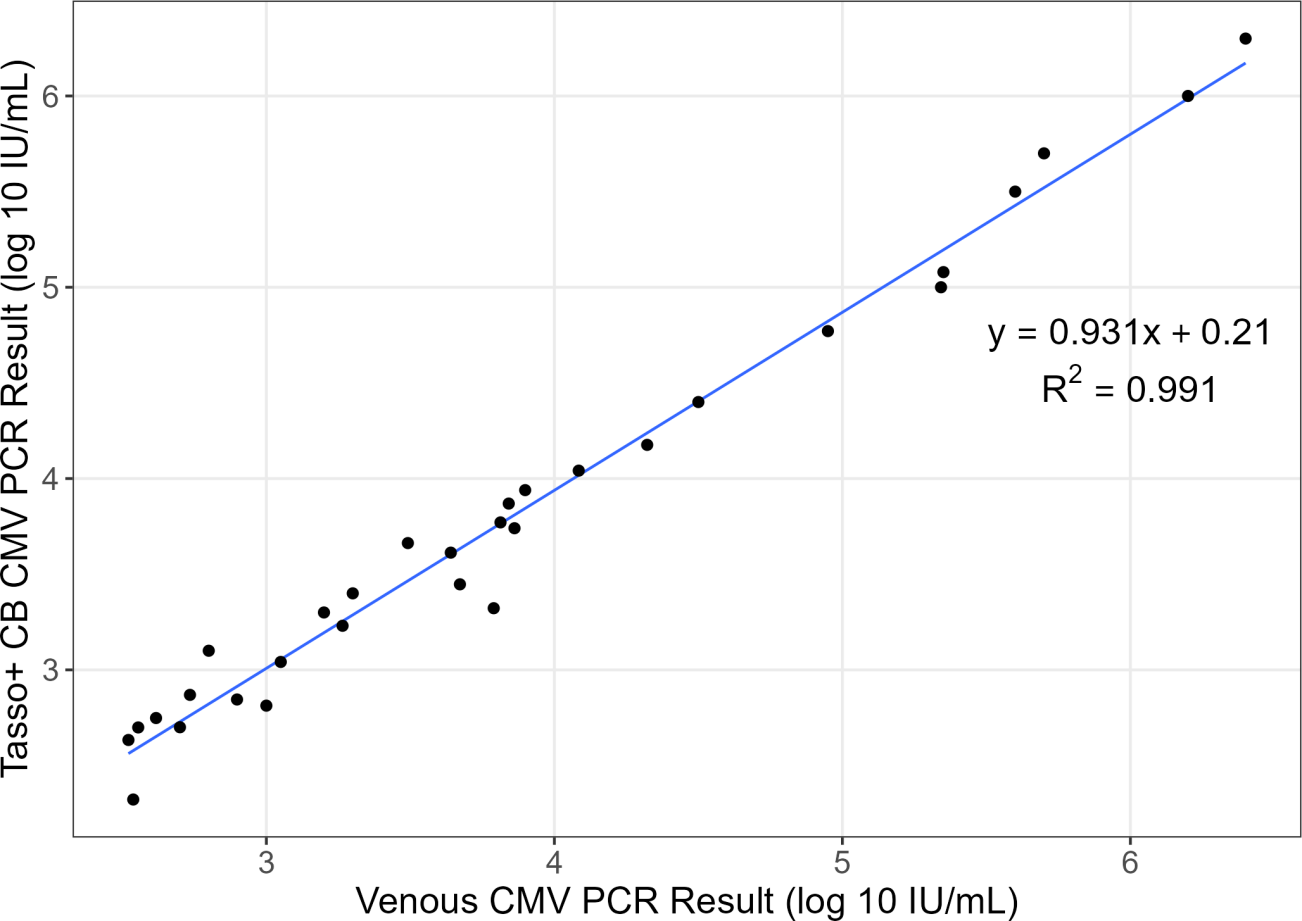
Scatter plot and linear regression results for samples that yielded a quantifiable CMV DNAemia result by both methods.

**Fig 3:**
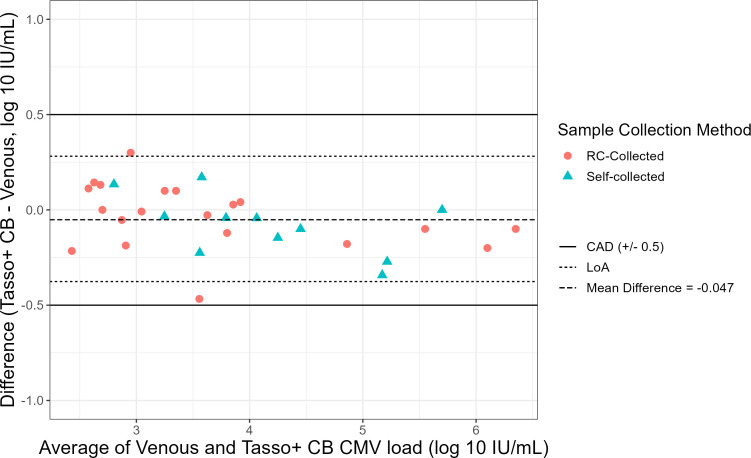
Bland–Altman plot comparing CMV viral load measured in venous blood and Tasso+ CB, respectively. The limits of agreement (LoA) represent the estimated interval in which the bias of 95% of sample pairs will lie and fall within the *a priori* selected clinically accepted difference (CAD) of ± 0.5 $\log_{10}CMV\frac{IU}{mL}$ .

**Fig 4:**
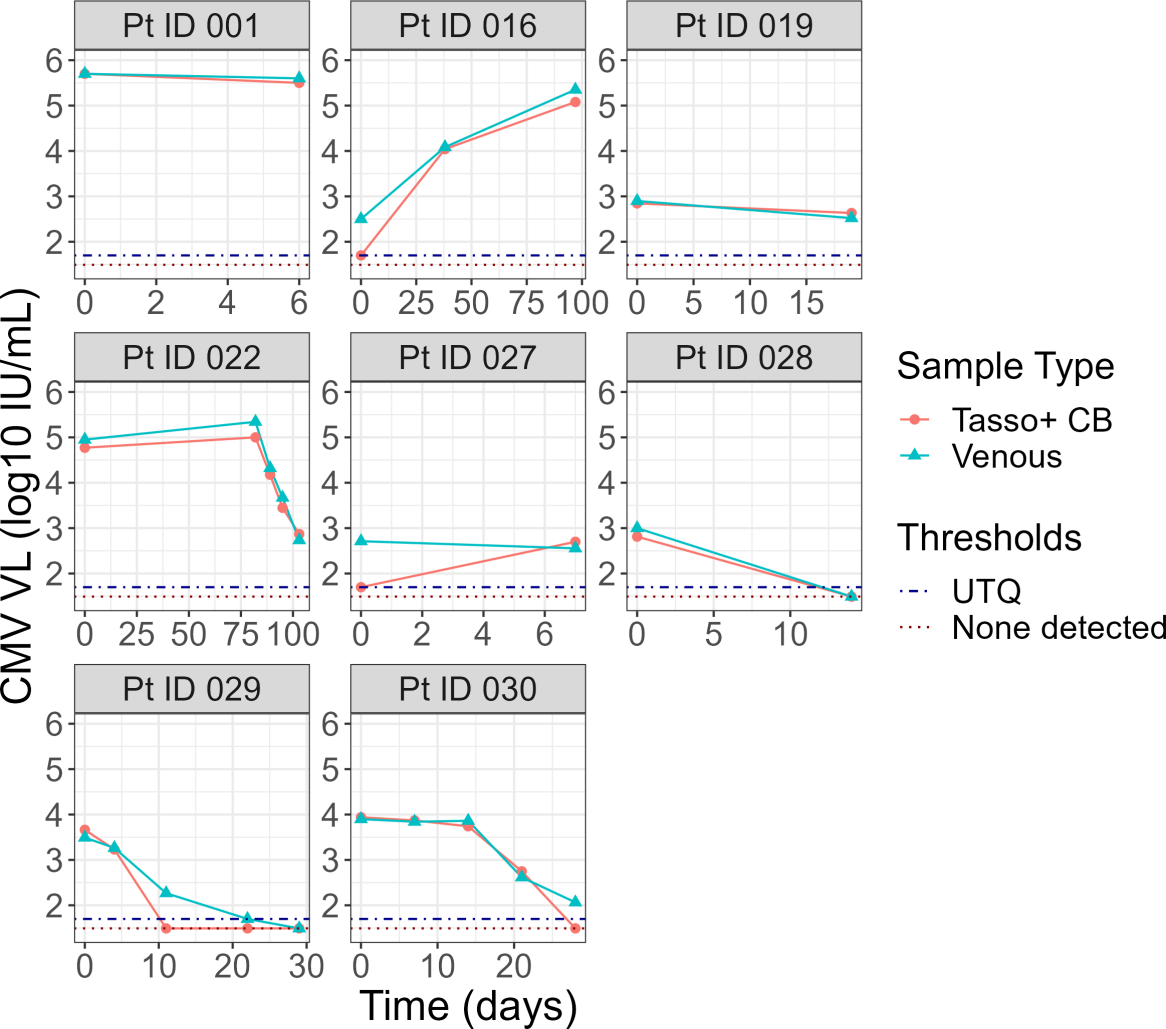
Longitudinal analysis of sequential CMV viral load measurements in both Tasso+ CB and venous samples. Unable to quantitate (UTQ) represents samples that were detected >LOD but <LLoQ were therefore not quantifiable. None detected represents samples in which no CMV was detected (<LOD).

Among the 31/44 (70%) of samples that yielded a quantifiable result (>LLoQ) by both methods, there was an excellent correlation between the two methods in quantitation of CMV DNAemia ($R^{2}=0.99$, Fig. 2). The CCC among these samples was 0.988, indicating an excellent agreement between the quantitative results of CMV DNAemia measured by both methods. CCC was similar between self- and RC-collected samples (0.980 vs 0.989). To further assess agreement in quantitative CMV DNAemia viral loads between methods, we conducted a Bland–Altman analysis using an *a priori* CAD of $\pm 0.5\;log_{10}\frac{IU}{mL}.$ We identified a mean bias of −0.047 $\log_{10}\frac{IU}{mL}$, with limits of agreement of −0.38 to 0.28 $\log_{10}\frac{IU}{mL}$, which was well within the CAD (Fig. 3). CMV DNAemia levels in Tasso+ CB samples were consistently lower than venous samples at viral loads >4.0 $\log_{10}\frac{IU}{mL}$ compared with samples with CMV DNAemia <4.0 $\log_{10}\frac{IU}{mL}.$ Visually, biases did not differ by collection method. Eight participants provided serial samples (26 samples; mean, 3.25 samples per patient) to assess changes in CMV DNAemia during treatment. Visually, there was a high correlation in CMV DNAemia levels between methods in serial samples during treatment (Fig. 4). We determined whether there was a significant relationship between time and the difference in CMV VL measured in venous and Tasso+ CB by two methods. First, we conducted a paired-sample *t*-test of the difference in CMV VL at the first and last time points for each participant. The mean difference in CMV VL between the Tasso+ CB and venous samples was not significantly different between the first and last time points (*P* = 0.36). Second, we included time as a parameter by regressing the difference in CMV VL between methods on the number of days following enrollment for all patients with sequential samples. Time was not a significant variable in the linear model (*P* = 0.83).

### Method acceptability

Of 30 (47%) participants, 14 completed acceptability surveys. The survey assessed discomfort associated with the Tasso+ device and venipuncture, the ease and confidence of using the Tasso+ device, and preference between Tasso+ and venipuncture for future blood collection (if theoretically available as an option). Among the participants who completed the survey, the discomfort of both venipuncture and Tasso+ was similarly low with a median (IQR) discomfort rating of 1 ± 1 and 1 ± 0, respectively. Of 14 participants who filled out the survey (50%), seven self-collected a specimen with the Tasso+ device and rated it 4 ± 1 in ease of use of the device. Of 14 participants (86%), 12 answered the final two survey questions. Of 12 participants (83%), 10 were confident in self-collection with the Tasso + device in the future (four strongly agreed and six agreed), and the other two neither agreed nor disagreed. Of 12 participants (92%), 11 expressed a preference for using the Tasso+ device for future blood collection compared with venipuncture if available as an option (four strongly agreed and seven agreed), and one participant neither agreed nor disagreed.

## DISCUSSION

We describe a convenient, patient-acceptable, and relatively sensitive methodology (Tasso+ device) for collection of a liquid CB sample for measurement of plasma CMV DNAemia. An important advantage of the technology over other CB collection methods, such as dried blood spots, is that a liquid sample is collected that could facilitate more accurate quantitative measurement of multiple analytes (expressed per unit blood volume). The relative simplicity and patient acceptability of the device facilitate patient blood self-collection and could facilitate patient-empowered testing as a means to improve adherence for laboratory monitoring. In theory, this collection method could be applicable for monitoring other viruses that are routinely serially assessed in the blood of transplant and other selected patient populations (e.g. CMV, EBV, BK virus, etc.), but this should be assessed in future studies. The potential applications of this technology are broad and include use in resource-limited settings or field research studies, situations of limited access to phlebotomy facility, patients with communicable diseases, or for patients unwilling to undergo venipuncture. Preliminary studies have demonstrated good concordance between CB samples collected with Tasso+ devices and venous samples for multiple analytes (5, 6, 13–15).

The estimated LoD and LLoQ of the Tasso+ methodology were approximately 308 and 588 IU/mL, respectively, and were similar to a previously published study of dried blood spots (8) but higher than with a standard venous sample. Nevertheless, this level of sensitivity is likely sufficient for many clinical purposes in transplant patients (1, 16, 17). Because a formal sensitivity analysis was not feasible in the current study, additional studies are required to more precisely define these parameters of the Tasso+ methodology. The lower sensitivity likely reflects the smaller blood volume collected by the Tasso+ device in some draws (typically ~0.5 mL), and the corresponding dilution needed to achieve the required input volume for the specific quantitative CMV PCR assay used in the study. Pre-warming of the area for Tasso+ device placement and other approaches to optimize the volume of collection should be assessed in future studies. The kinetics of CMV DNAemia during the course of treatment was well correlated in sequential samples, suggesting the utility of the Tasso+ methodology for monitoring response to treatment, in addition to cross-sectional testing. The Tasso+ collection device is further well-suited for patient blood self-collection based on the relative simplicity of use and the favorable patient acceptability survey responses in our study. Future studies should directly compare Tasso+ with other CB self-collection methods (e.g. dried blood spots) to determine whether there are consistent patient preferences for a given methodology.

The strengths of our study include the prospective study design with paired venous and Tasso+ CB specimens, inclusion of sequential samples to assess changes in CMV viral load during treatment, and the use of a well-studied and commercially available quantitative CMV PCR assay. Limitations include the moderate sample size, relatively few samples that were patient self-collected (vs. RC-collected), and inability to analyze concordance in patients who were not receiving anti-CMV therapy. The potential impact of delayed separation of plasma from whole blood was not assessed because the Tasso+ CB sample was immediately centrifuged. However, previous studies suggest that the viral load in plasma samples is not affected by delayed separation for up to 72 h (18, 19), which is anticipated to accommodate the delay in separation imposed by overnight shipped samples. Future studies should be conducted to confirm the lack of effect of delayed plasma separation on CMV viral loads in Tasso+ CB samples and verify that patient self-collected samples are comparable to study personnel-collected samples. Additionally, for home self-collected samples, factors, such as the effect of transport times during ambient shipping, would need to be formally assessed before implementing the method for clinical care. Even if this technology is ultimately validated for home use, the additional complexity for the laboratory (non-standard tube, low sample volume) will need to be carefully considered.

In summary, we have demonstrated that CMV DNAemia can be accurately assessed in CB collected with the Tasso+ device and that the results are generally comparable to venous plasma specimens but with lower sensitivity. Future studies with larger sample size that include patient self-collection in off-site (home or other) locations should be conducted to further evaluate this approach.

## SUPPLEMENTAL MATERIAL

Supplemental material spectrum.00030-24-s0001.docx Patient satisfaction survey provided to study participants.

ASM does not own the copyrights to Supplemental Material that may be linked to, or accessed through, an article. The authors have granted ASM a non-exclusive, world-wide license to publish the Supplemental Material files. Please contact the corresponding author directly for reuse.
