# Supplementary material for: Evaluation of the Tasso+ blood self-collection device for quantitation of plasma cytomegalovirus (CMV) DNAemia in adult solid organ transplant recipients (SOTr)
Source: Microbiol Spectr. 2024 May 21;12(7):e00030-24. doi: 10.1128/spectrum.00030-24 (PMC11218524; doi:10.1128/spectrum.00030-24)
Supplement: Supplemental material — Patient satisfaction survey provided to study participants. [file spectrum.00030-24-s0001.docx]

**Appendix 1: Patient satisfaction survey**

**University of Washington**

**Clinical Performance of the Tasso Blood Collection Device in the**

**Management of Solid Organ Transplant Patients**

**Patient Satisfaction Survey: On-site Visit**

Subject: TASS-____

Survey date: ___ ___ /___ ___ /___ ___ ___ ___ (mm/dd/yyyy)

|  | **No Discomfort Moderate Severe Discomfort** | | | | |
| --- | --- | --- | --- | --- | --- |
| Please rate your discomfort level associated with venipuncture. | **1** | **2** | **3** | **4** | **5** |
| Please rate your discomfort level associated with the Tasso device | **1** | **2** | **3** | **4** | **5** |
|  | **Very Difficult Very Easy** | | | | |
| **For patients who self-collected:**  Please rate the ease of use of the Tasso device. | **1** | **2** | **3** | **4** | **5** |
|  | **Strongly Disagree** | **Disagree** | **Neither Agree nor Disagree** | **Agree** | **Strongly Agree** |
| I feel confident collecting my own blood samples using the Tasso device. |  |  |  |  |  |
| If I need blood drawn in the future, I would prefer the Tasso device over venous draw at the lab. |  |  |  |  |  |

What did you like about Tasso blood collection? Please comment below.

_______________________________________________________________________________________________________________________________________________________________________________________________________________________________________________________________

What did you not like about the Tasso blood collection? Please comment below.

_______________________________________________________________________________________________________________________________________________________________________________________________________________________________________________________________
